# Development and Validation of the Prevention of Toxic Chemicals in the Environment for Children Tool: A Questionnaire for Examining the Community's Knowledge of and Preferences Toward Toxic Chemicals and Children's Brain Development

**DOI:** 10.3389/fpubh.2022.863071

**Published:** 2022-05-11

**Authors:** Rivka Green, Bruce Lanphear, Erica Phipps, Carly Goodman, Jasmine Joy, Samer Rihani, David Flora, Christine Till

**Affiliations:** ^1^Department of Psychology, York University, Toronto, ON, Canada; ^2^Faculty of Health, Simon Fraser University, Burnaby, BC, Canada; ^3^Prenatal Environmental Health Education (PEHE) Collaboration, University of Ottawa, Ottawa, ON, Canada

**Keywords:** children's environmental health, prevention, toxic chemicals, brain development, scale development

## Abstract

Early-life exposures to toxic chemicals can adversely impact brain development. Understanding people's knowledge of the impact of toxic chemicals on brain development is critical to reduce widespread exposure to chemicals. Yet it is unknown what people know about risks of toxic chemicals and how to reduce exposures. We developed and validated the questionnaire, *PRevention of Toxic chemicals in the Environment for Children Tool (PRoTECT)*, to examine people's knowledge and attitudes about the influence of toxic chemicals on child development. We used best practices for developing and validating scales. First, we drafted items to assess knowledge of the impact of toxic chemicals on brain development, levels of concern regarding exposures, and preferences for prevention of neurodevelopmental disorders. Second, we received feedback on item clarity from five focus groups consisting of 46 community participants. In addition, 17 experts completed a content validity scale for each item and provided qualitative feedback. We administered the revised 18-item questionnaire to 190 participants of child-bearing age for scale development, and using exploratory factor analysis, we found evidence for a four-factor model of *PRoTECT*, RMSR = 0.05, of which 16 of the 18 items had adequate content validity with loadings >0.40 on a derived factor. We discuss future directions and applications of *PRoTECT*.

## Introduction

Toxic chemicals are an insidious threat to children. Toxic chemicals elevate the risk for neurodevelopmental disorders, including learning disabilities, attention deficit hyperactivity disorder (ADHD), and autism spectrum disorder (ASD) (1). The developing brain is particularly vulnerable to toxic chemicals, even at low doses that might not have an adverse effect on adults. Therefore, early identification and recognition by the public of potential sources of exposure to toxic chemicals are crucial to protect children. An extensive number of studies have examined the impact of early life environmental exposures on brain-based disorders, but few have examined ways to prevent or of reduce exposure. Further, little is known about what the public knows about the impact of toxic chemicals on child development, or how to educate practitioners and parents to recognize toxic chemicals and protect children.

Over the last two decades, scientists and physicians have called for increased education on children's environmental health in the healthcare setting (2–5). In 2021, the American College of Obstetricians and Gynecologists (ACOG) called for obstetrician-gynecologists and other prental health providers to become more knowledgeable about the impact of toxic chemicals on prenatal health to conduct an environmental health history, provide information on risks, and refer their patients to experts when indicated (6).

Education of parents and pregnant women has received less attention than healthcare providers, even though mothers are eager for information about pregnancy and early childhood (7). In 2009, Grason and Misra were unable to identify any peer-reviewed articles on women's knowledge about toxic chemicals in pregnancy, and highlighted strategies to address the knowledge gap on toxic chemicals during pregnancy, including enhancement of news media, product labeling, and promotion of health-care provider counseling for women and parents (8). Despite this, Crighton et al. found that a large proportion of mothers felt inadequately educated on environmental health risks, and many did not perceive exposure to toxic chemicals in the indoor environment to be of high concern (9). Yet, Laferriere et al. found that mothers who were concerned about environmental health risks were more than twice as likely to engage in protective behaviors to reduce exposures, such as opting for organic foods or switching to safer household products, suggesting that parental concern may play a role in behavior modification (10).

In fact, several studies have shown that educating parents about the impacts of toxic chemicals on children's health can influence their behaviors. One recent American study found that greater parental concern about toxic chemicals was associated with lower urinary concentrations of phthalates and phenols in children's urine (11). Another American study showed that expectant mothers' exposure to media coverage about the impact of pesticides, arsenic, and bisphenol A on children's health was associated with self-reported intent to reduce exposure to toxic chemicals, especially during pregnancy (12). Subsequent studies stressed the role of mass media in providing environmental health information to new and expectant mothers. Specifically, encountering online media articles on prenatal and child health was strongly associated with increased perceptions of personal accountability for choices that optimize environmental health (13). Similarly, Barbir et al. found that respondents' willingness to reduce plastic consumption was associated with their access to information (14). Furthermore, Crighton et al. indicated that mothers were willing to change their behaviors and reduce exposures when they were educated about the risks and actions to take (9). Taken together, these findings suggest that awareness and education of the impacts of toxic chemicals are key for reducing exposures to toxic chemicals.

Acknowledging the impact of toxic chemicals on brain development may help prevent neurodevelopmental disorders by encouraging the public to find ways to reduce widespread exposure to chemicals and advocate for stricter regulations to prevent chemicals from entering the environment. While advocations for regulatory policies on the widespread commercial use of toxic chemicals continues, families can be educated on how to make safer choices for their children's health. Yet, it is unknown what people know about the risks of toxic chemicals, and with the absence of information on their knowledge, it is unknown how clinicians and researchers can better support parents. While people may not understand the risks, or how to manage exposures, information disseminated by the chemical industry about the safety of their products further undermines public awareness of the scientific evidence of developmental health risks associated with toxic chemicals. Lastly, documenting parents' preferences for prevention of neurodevelopmental disorders may also accelerate the development of regulations and enhance parental advocacy.

We designed and validated the *PRevention of Toxic chemicals in the Environment for Children Tool (PRoTECT)* to assess parents' knowledge about toxic chemicals and brain development. We also wanted to assess their level of concern about toxic chemicals and preferences for the prevention of neurodevelopmental disorders.

## Methods

We developed and validated *PRoTECT* using best practices for developing and validating scales (15). The development and validation of the survey involved three phases: (1) item development, (2) scale development, and (3) scale evaluation. Our study received ethical approval from the York University ethical review board.

### Item Development

#### Domain and Item Generation

The questionnaire's domains were developed and defined based on the authors' (RG, BL, EP, CT) collective expertise in children's environmental health, collaboration with stakeholders and funders, and a literature review to identify areas that were lacking in the field. We identified three domains of interest: (1) knowledge of developmental neurotoxicity, (2) level of concern about exposure to toxic chemicals, and (3) preferences for the prevention of neurodevelopmental disorders.

To define the domains of interest and generate items, we conducted a scoping review using the following key terms: “toxic chemicals” OR “toxi^*^” OR “chemicals” OR “environment^*^” AND “neurodevelopment” OR “ADHD” OR “autism” OR “cogniti^*^” OR “behav^*^” AND “attitude” OR “knowledge” OR “preference”. After identifying key papers on this subject, we used the references from the papers to identify additional relevant articles. The scoping review only identified nine empirical studies on parents' and pregnant women's knowledge and attitudes of children's environmental health. Thus, we extended our search to include healthcare providers' knowledge of developmental neurotoxicity.

The first domain, knowledge of developmental neurotoxicity, was defined as knowledge of the relation between toxic chemicals and neurodevelopmental outcomes. Specifically, this domain included knowledge of the impact of *low levels* of toxic chemicals on brain development, the ubiquity of toxic chemical exposure present in the environment and everyday products, children's and fetuses' unique vulnerability to toxic chemicals, and the government's oversight and regulation of toxic chemicals. The second domain, level of concern, was defined as people's perceptions of the risks associated with exposure to toxic chemicals, including where they are found, whom they trust to provide information on toxic chemicals, and whether reducing exposure is important. The final domain, attitudes toward prevention, included people's preferences toward prevention relative to treatment of neurodevelopmental disorders, their knowledge of the government's expenditures to prevent neurodevelopmental disorders, their preferences, if any, for the government to prioritize funding into prevention, and their understanding of the prevention paradox (i.e., more children would benefit by preventing neurodevelopmental disorders than from treating disorders once they occur).

No validated scales have examined parental knowledge of developmental neurotoxicity and preferences for prevention, so we used the articles obtained in the scoping review to develop phrasing and rating scales. For example, we looked at terms that refer to “toxic chemicals” and found that studies have used “environmental exposures” (5), “environmental toxicants” (16), “chemical molecules” (17), or “endocrine-disrupting chemicals” (18). Some studies assessed answers qualitatively through thematic analysis (17, 18) and others used Likert scales (5, 16, 19).

#### Content Validity

Using previous studies, we generated items with a goal of five items per domain for a total of 15 items. Our group revised the items four times prior to external review. Our final compilation included 19 items. We chose to use a five-point Likert-type response scale, ranging from 1 *(strongly agree)* to 5 *(strongly disagree)* based on previous research of health-related knowledge surveys (5, 16, 19).

We assessed content validity by having experts evaluate the questionnaire items and using focus groups of the target population. We contacted 20 international experts in epidemiology, psychology, environmental health advocacy, neurodevelopment, and public education to assess the content validity of questionnaire items and provide feedback using a Google Survey platform. Each expert was asked to rate the content relevance of each item using a four-point response scale [i.e., 1 (*not relevant*), 2 (*unable to assess relevance without revision*), 3 (*relevant but needs minor revision*), and 4 (*very relevant and succinct*)]. Following each item, experts were asked to provide general qualitative feedback on the item and specific qualitative feedback about how it was worded. At the end of the questionnaire, experts provided their general impressions on key words used throughout the survey, as well as whether any content was missing or any item should be removed.

We conducted three rounds of expert reviews with 17 of the 20 experts (85% response rate). Following each round of expert review, we executed content analysis to determine the proportion of agreement among the experts. We calculated a content validity index (CVI) for each item based on the proportion of experts who rated the item as content valid (rating of three or four). After the final expert review, we calculated a CVI for the entire instrument based on the proportion of questionnaire items that received a content valid rating.

While the expert reviews were underway, we conducted five focus groups across North America, with a total of 35 participants (69% female, age range: 18–45), to assess the clarity and relevance of questionnaire items. Participants were recruited through social media postings (Facebook, Instagram, Twitter) and email. All focus groups were held on the video conferencing platform, Zoom, for ~1 h. Four focus groups were conducted with both parent and non-parent participants to assess item accessibility and comprehensibility. The fifth focus group was conducted with parents only, to assess emotional reactions to the survey items. All participants consented to be a part of the focus groups and received a $25 gift certificate for their involvement in the study.

The focus groups were conducted as semi-structured cognitive interviews to assess item interpretation and facilitate open discussion. After reading each questionnaire item, participants were asked open-ended questions including “how would you phrase this item in your own words?”, “is there anything in this statement that you would change?” and “do you have any reactions when reading this item?”. We also asked questions specifically tailored to each item with a similar open-ended format based on comments and suggestions from the expert reviews.

The focus groups were transcribed and coded to collect qualitative and quantitative data. One research assistant conducted the interview (SR), two research assistants transcribed and coded all focus groups (CG, JJ), and the lead author observed all groups and prepared the questions and guided the discussion using the chat function (RG). The research assistants transcribed the groups independently and collated their transcripts following the final focus group. Qualitative information was collected using the collated transcript to assess descriptive judgment and participants' emotional responses to items. We created a coding matrix to collect quantitative information and to determine the number of verbal and non-verbal cues indicating agreement or dissent with questions (20, 21).

After three rounds of expert reviews and five focus groups, we compiled the responses to inform new items on *PRoTECT* which was presented to our internal team for dialogue and feedback (described in more detail in results).

### Scale Development

#### Sampling and Survey Administration

We used the next version of *PRoTECT* to collect data for a preliminary exploratory factor analysis. We recruited participants through social media postings (Facebook, Instagram, and Twitter) and offered them a $5 gift card once they completed the survey. *PRoTECT* was administered *via* Qualtrics, an online survey platform. Following consent, participants were presented with a brief demographic questionnaire, followed by a general introduction to *PRoTECT*, and then the questionnaire items. The items were presented such that a participant could only view one item at a time and could not return to previous items. All responses were timed to ensure participants' responses were valid (i.e., they spent sufficient time filling out the questionnaire).

### Scale Evaluation

#### Statistical Analyses

The items' five-point Likert-type response scale suggests that a traditional linear factor analysis model may not be appropriate for the resulting ordered, categorical data; accordingly, we fit exploratory factor analysis (EFA) models to the polychoric correlations among items using unweighted least squares estimation [see (22)] and fit a linear EFA model using Pearson product-moment correlations as a sensitivity analysis. Prior to estimating EFA models, we examined the polychoric and product-moment associations among items. We used a scree plot, parallel analysis, and root mean square residual (RMSR) statistics to identify the optimal number of factors for the EFA model [see (22)].

## Results

### Results From Expert Review

#### Quantitative Results

The first round of expert review consisted of six experts in epidemiology, psychology, children's environment health advocacy, and medicine. In the first round, 17 of 18 items had excellent content validity ratings (CVI ≥ 0.83). The second round consisted of six additional experts and 17 of 19 items (one item was added based on results from the first round) had excellent content validity ratings (CVI ≥ 0.83). Two items were dropped in the second round based on low ratings. In the third and final round of expert review, four experts participated, and 13 of 17 items had excellent content validity ratings (CVI = 1.00). The CVI of the total questionnaire in round three was 0.76 (see Table 1 for the CVI scores for each item in each round). Items that did not receive an adequate CVI were reworded, reordered, or deleted in accordance with experts' qualitative feedback and recommendations from focus group participants.

**Table 1:** The number of experts with a content valid rating and the calculation of CVI for each item.

**Item**	**Round 1**		**Round 2**		**Round 3**	
	**Number of experts**	**CVI**	**Number of experts**	**CVI**	**Number of experts**	**CVI**
1. Toxic chemicals in our day-to-day lives, like air pollution or lead in drinking water, can increase a child's risk of developing ADHD or autism	6	1.00	6	1.00	4	1.00
2. The amount of resources that my government invests to prevent learning and behavioral conditions in children is about equal to the amount it invests to treat these conditions	6	1.00	5	0.83	3^*^	0.75
3. My government should devote more resources to make sure that consumer products do not contain toxic chemicals that are unsafe for children.	5	0.83	6	1.00	4^*^	1.00
4. Of all the things my government does to keep children healthy, reducing children's exposure to toxic chemicals should be a priority.	5	0.83	5	0.83	N/A	N/A
5. There are things parents can do during pregnancy and early childhood to reduce their child's risk of developing a learning or behavioral condition, like ADHD or autism.	6	1.00	5	0.83	N/A	N/A
6. The levels of toxic chemicals commonly found in food, consumer products, and drinking water are too low to interfere with children's brain development.	6	1.00	5	0.83	3^*^	0.75
7. Most governments spend 95% or more of their budgets to treat disease and disabilities. Governments should devote more of their budget to prevent these conditions	5	0.83	6	1.00	4^*^	1.00
8. I trust scientists' recommendations about how to reduce exposure to toxic chemicals	4	0.67	4	0.67	4^*^	1.00
9. Children are more likely to be harmed by toxic chemicals than adults, especially before they are born.	6	1.00	6	1.00	4^*^	1.00
10. I trust companies to make products that don't contain harmful chemicals.	6	1.00	6	1.00	4^*^	1.00
11. If I knew how to reduce children's exposure to toxic chemicals, I would do it.	6	1.00	4	0.67	4^*^	1.00
12. The number of children who would benefit from regulating toxic chemicals linked to learning and behavioral conditions is greater than the number of children who benefit from treating these conditions.	6	1.00	5	0.83	3^*^	0.75
13. I want to learn more about how to reduce children's exposure to toxic chemicals.	6	1.00	6	1.00	4	1.00
14. Toxic chemicals are found in everyday products, including foods, cleaning products, and personal care products.	6	1.00	6	1.00	N/A	N/A
15. All parents have equal opportunities to protect their children from toxic chemicals, regardless of income level, race or where they live	6	1.00	5	0.83	3^*^	0.75
16. My government has regulations to make sure that personal care products, furnishings, and food do not contain harmful levels of toxic chemicals.	6	1.00	6	1.00	3^*^	0.75
17. If toxic chemicals were a threat to my family's health, my pediatrician, obstetrician, or general practitioner would have told me about it.	6	1.00	6	1.00	4^*^	1.00
18. Research shows that most pregnant women have toxic chemicals in their blood	5	0.83	6	1.00	4^*^	1.00
19. Toxic chemicals that pregnant women are exposed to can increase the risk of their child having a learning or behavioral condition after they are born.	N/A	N/A	5	0.83	4^*^	1.00
20. I try to purchase products that do not contain toxic chemicals that may be harmful to my family	N/A	N/A	N/A	N/A	4	1.00

* *Indicates that the wording of the item was changed in subsequent rounds to reflect changes posed by expert and focus group participants. See Supplementary Table 2 for more information on this*.

#### Qualitative Results

In addition to providing a rating between 1 and 4, experts provided qualitative feedback for each item and their general impressions at the end of the questionnaire. We aggregated the individual comments for each item, and if two or more experts made a similar comment, we addressed that comment and restructured the item. For example, many experts felt that an item (i.e., see item 5 on rounds 1 and 2) could elicit blame in parents, suggesting that “[we] need to be very careful not to infer ‘blame' in this type of query” and to “take care not to induce potentially unproductive feelings of guilt in parents”. Therefore, the item was restructured to reduce its potential to incite guilt in parents (i.e., see item 5 on round 3). In other examples, experts rephrased items in their own words to reduce jargon or added words to make the item more specific. For example, an expert suggested adding the word “effective” before regulations (see item 4 on round 3).

The experts' general impressions revealed several themes. First, experts reported that certain terms (i.e., “toxic chemicals” or “learning and behavioral conditions”) needed to be defined at the beginning of the questionnaire. For example, one expert wrote, “I would try to use as widely accessible terminology as possible. I think that may mean having a few lead-in comments that help to define terms that are going to be used in subsequent questions”. Another theme that emerged was whether some items may be leading to get an “agree” response. One expert wrote, “wonder if some questions may be leading” and another wrote, “seems like it is loaded to get an ‘agree' response”. Thirdly, experts reported that many items had “too many clauses” in it or “lots of nuances” that would make it difficult for a parent to understand. Lastly, experts expressed support for our project and shared that it is “so great that this is being done!!!”.

We also asked experts if any content was missing. If two or more experts suggested the same item, we considered adding it to the survey. This process led to a new item asking about genetics vs. environmental exposures in determining health outcomes. For example, one expert suggested “do respondents perceive developmental disorders as random or pre-determined (e.g., genetic)?”. Two experts suggested adding items on “pesticides” and “labeling like natural vs. organic”. Experts also suggested adding what parents are “already using to minimize chemical exposure or what strategies they currently think are helpful” as well as “what entity respondents trust to be effective at ensuring the safety of their food, consumer products, etc”. Still, most experts did not feel any content was missing. One expert wrote “one can always think of other questions, but I think the survey hits the right mark and is a good length”.

### Results From the Focus Groups

The purpose of the focus groups was to understand participant's grasp of phrases or terminology of the items, as well as their preference. For each item, we coded focus group transcripts to indicate participant agreement, dissent, and significant statements suggesting either agreement or dissent (Supplementary Table 1). For example, when asked whether participants preferred the use of “toxic chemicals” in Item 1 instead of “environmental chemicals”, 11 participants indicated assent, five dissent, one provided a significant statement indicating agreement, one provided a significant statement indicating dissent, four indicated no preference, and seven did not respond. The questionnaire items were then edited to reflect participant feedback.

Participants provided qualitative feedback for each item and their general impressions. Many themes overlapped with the expert reviews. For example, participants shared that some of the items elicited blame or guilt. In reference to item 5 referenced above, one participant mentioned “this felt like a moment of blame”, and another said, “[there is] something triggering in this question for me as a parent”. Participants also indicated that certain wording was too technical. For example, with respect to item 12 on rounds 1 and 2 (i.e., trying to explain the prevention paradox), one participant “was wondering if we could break it down because I had some trouble understanding”, another mentioned that “people who aren't involved in science may be confused”, and a third participant said “it took me a few times and I'm not sure if I even understand it right”. Participants also expressed that they wanted the items to be specific and not too vague. For example, in reference to item two (rounds 1 and 2), one participant asked, “can we be more specific what ‘more resources' means”, and another said “resources is very broad…could be almost anything”. When asked whom people would trust to get this information in item eight (rounds 1 and 2), participants expressed that they would want the information coming from scientists who study the topic. For example, one participant said, “I would overall trust a health professional or scientist in a specific field”. In some instances, participants suggested rewriting the item altogether. One participant said, “I would suggest rewording the whole thing”.

**Figure 1:**
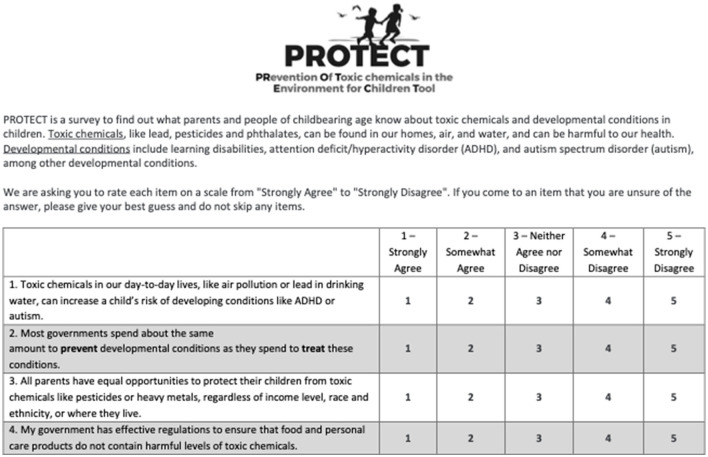
Final version of PRoTECT.

Participants said that they wanted key definitions before terms that would be used several times (i.e., “toxic chemicals” and “developmental conditions”). For instance, participants asked “can we add in brackets what these learning and behavioral conditions might be” and “I'd like to know a bit more about what the toxic chemicals are”. Thus, we added key definitions to the beginning of the measure (Figure 1). Similar to the expert review, participants shared the overall importance of the survey, and emphasized that this work is “…so important to research and get more data”, that it is a “really important topic”, and that it “…doesn't veer away from what the objective is… clear and succinct”. Some participants said they hadn't heard a lot on this topic.

Overall, qualitative results revealed good content validity (“I would describe it as a survey about toxic chemicals and child health and development”), and the Likert-type response was understood (“the nature of the questionnaire is to agree or disagree… I know that it's not a statement, it's asking for your opinion”).

#### Item Reduction

Items underwent several rounds of revision based on expert and focus review. One item (item 4 on rounds 1 and 2) was removed due to poor ratings from both experts and focus group participants. The version of *PRoTECT* administered to participants consisted of 18 items and included a brief introduction to define key terms (Supplementary Figure 1).

#### Results From EFA

A total of 235 participants were recruited and began the *PRoTECT* questionnaire. Of those, 190 (81%) completed the questionnaire [55% female, mean (SD) age = 26.32 (5.40)], providing a ratio of over 10 participants per item.

The strength of the inter-item polychoric correlations ranged from approximately zero (*r* = 0.02) to *r* = 0.65. To determine the optimal number of factors to explain the pattern of inter-item correlations, we first plotted the eigenvalues of the correlation matrix with a scree plot, which suggested that four factors account for the pattern of inter-item correlations (i.e., there is a noticeable decrease after the fourth eigenvalue, after which the remaining eigenvalues level off more steadily).

A parallel analysis using 100 iterations also suggested four factors. The RMSR for the 4-factor model (i.e., RMSR = 0.05) was substantially better than the RMSR from the 3-factor model (RMSR = 0.08) or 2-factor model (RMSR = 0.10). Therefore, we present the results from the four-factor model with an oblimin rotation of the factor pattern matrix (Table 2). Importantly, 16 out of 18 items had factor loadings above 0.40 on one of the four factors. Item 18 had factor loadings of 0.35 on two factors. Item 11 did not have a substantial loading on any factor. Correlations among factors are shown in Table 3.

**Table 2:** Factor loadings of the four-factor model.

	**Factor 1**	**Factor 2**	**Factor 3**	**Factor 4**
Item 1	−0.03	0.24	**0.76**	−0.26
Item 2	0.08	0.20	−0.06	**0.55**
Item 3	0.03	0.30	−0.06	**0.66**
Item 4	0.03	**0.67**	0.01	0.01
Item 5	−0.02	−0.14	**0.88**	0.04
Item 6	0.32	0.08	**0.43**	0.17
Item 7	−0.12	**0.62**	0.05	0.14
Item 8	0.21	−0.07	**0.57**	0.24
Item 9	**0.71**	0.00	0.09	0.11
Item 10	**0.61**	−0.23	0.08	0.22
Item 11	0.19	−0.07	0.05	−0.06
Item 12	0.10	**0.66**	−0.05	0.21
Item 13	**0.40**	0.08	0.21	−0.05
Item 14	**0.47**	−0.11	0.09	0.11
Item 15	**0.76**	0.05	0.05	0.01
Item 16	**0.82**	0.09	−0.09	−0.08
Item 17	**0.57**	−0.09	−0.09	−0.31
Item 18	0.35	0.35	0.08	−0.41

*Values in bold denote loadings >0.40*.

**Table 3:** Factor correlations.

	**Factor 1**	**Factor 2**	**Factor 3**	**Factor 4**
Factor 1	-	0.15	0.30	0.06
Factor 2		-	0.04	0.08
Factor 3			-	−0.06
Factor 4				-

#### Sensitivity Analysis

A four-factor model fitted to the inter-item product moment correlations also had an adequately low RMSR statistic = 0.04, and the oblimin-rotated factor pattern led to similar conclusions about how each item related to each of the four factors.

## Discussion

This study developed and described *PRoTECT*, a questionnaire assessing knowledge about toxic chemicals and neurodevelopment, level of concern, and preferences for prevention of neurodevelopmental disorders. The participants, including potential and current parents, said that the questionnaire was important and contained information that they do not hear about in their day-to-day lives. Similarly, expert reviewers indicated that this kind of research needed to be done and asked to be contacted with the results.

Feedback from several rounds of expert and participants generated a questionnaire consisting of 18 items. Considering this is the first questionnaire to measure knowledge and preferences surrounding toxic chemicals and brain development, we used factor analysis to provide meaning to the pattern of inter-item correlations and apply conceptual labels to the four factors described below.

Factor 1, which represented desire or intention to reduce exposure to toxic chemicals, included items such as “If I knew how to reduce children's exposure to toxic chemicals, I would try to do it” and “I want to learn more about how to reduce children's exposure to toxic chemicals”. Factor 2 represented trust in authority sources about exposure to toxic chemicals, and included items such as “My government has effective regulations to ensure that food and personal care products do not contain harmful levels of toxic chemicals” and “If toxic chemicals were a threat to my family's health, my pediatrician, doctor, or health care provider would have told me about it”. Factor 3 represented knowledge about developmental neurotoxicity (i.e., the relationship between toxic chemicals and development), and included items such as “Toxic chemicals in our day-to-day lives, like air pollution or lead in drinking water, can increase a child's risk of developing conditions like ADHD or autism” and “Reducing exposure to toxic chemicals during pregnancy and in early childhood can help lower a child's risk of developing a condition like ADHD or autism”. Lastly, Factor 4 represented knowledge about toxic chemicals and society, and included items such as “Most governments invest about the same amount to **prevent** developmental conditions as they spend to **treat** these conditions” and “All parents have equal opportunities to protect their children from toxic chemicals like pesticides or heavy metals, regardless of income level, race and ethnicity, or where they live” (Supplementary Table 3).

Since item 18 (“I am worried that my family may be exposed to toxic chemicals”) had a loading that was <0.40 and could be tapping more into emotional feelings (“worried”) as opposed to knowledge or attitudes, we removed it from the questionnaire.

Item 11 (“toxic chemicals are generally more harmful to babies and children than they are to adults”), a knowledge-based item that we felt was important to retain, did not have a substantial factor loading on any factor, indicating that it is not strongly influenced by any of the four constructs represented by the factors. Theoretically, it should be related to factor 3 (knowledge of developmental neurotoxicity); however, it could be phrased such that the item was not understood. For example, participants may have disagreed with the item if they thought it suggested that babies and children have more opportunity to be exposed to toxic chemicals, as opposed to its intended meaning of greater susceptibility to the adverse impacts of toxic chemicals. Considering that this item had been revised multiple times following focus groups and expert reviews, it could be that it is not self-explanatory. Therefore, to avoid confusion about opportunity for exposure between children and adults, we changed item 11 to: “*Exposure to toxic chemicals is particularly harmful to babies and children.”* The final version of *PRoTECT* can be found in Figure 1.

The highest inter-factor correlation was between factors 3 and 4 (*r* = 0.30), suggesting that people who knew more about developmental neurotoxicity were also more likely to want to reduce their exposure to toxic chemicals.

### Applications

*PRoTECT* can be used by researchers and clinicians to evaluate knowledge of toxic chemicals and neurodevelopment, as well as parents' and expectant parents' intentions to reduce their exposure to toxic chemicals and preferences toward prevention. Further, it can be used by researchers and children's environmental health advocates to assess understanding and urgency of the risks that toxic chemicals may pose, and to track knowledge and preferences over time.

Our intention is that *PRoTECT* will be used to inform and advance policy measures to reduce early-life exposures to toxic chemicals and, by extension, curb rising rates of neurodevelopmental disorders among children. In addition to limited research on the public's knowledge about the relationship between toxic chemicals and development, surprisingly little is known about people's preferences for healthcare resources going toward prevention of neurodevelopmental disorders, which is something we aimed to assess in the current study. Currently, little research and funding is geared to prevention of disease and disability. For example, one in four cases of childhood leukemia is attributed to toxic chemicals such as pesticides, solvents, and air pollution (23). Yet, only 4% of American health dollars are devoted to prevention of childhood cancers (24). Similarly, only 1% of the National Cancer Institute's budget for research in childhood cancer is devoted to prevention (24). While preference for prevention was not identified as a factor in our factor analysis, if we find that responses on *PRoTECT* reveal that parents prefer more funding go toward prevention of neurodevelopmental disorders, health sector stakeholders and decision makers can be informed of this preference. Parents can accelerate change by showing their preferences, as has been seen in other public health interventions, such as childhood vaccinations and water disaster in Flint, Michigan (25, 26). Knowledge of parental preference for more money and healthcare resources going toward prevention of neurodevelopmental disorders could accelerate change by presenting the information to political stakeholders.

### Future Directions

We are currently administering *PRoTECT* to a sample of 10,000 participants across five countries. We are conducting a nested, randomized-control trial (RCT) with inclusion of a knowledge translation tool in which half of the respondents will watch a video on the impact of toxic chemicals on brain development. The large sample will provide opportunities for further psychometric evaluation of *PRoTECT* and investigate whether the four-factor model of *PRoTECT* remains across a larger sample and more diverse group. A six-week follow-up survey will be conducted to assess differences in responses to *PRoTECT* after exposure to the video or its material, and whether any intent to change behaviors persist or increase after completing the questionnaire.

While we await stronger legislation to reduce toxic exposures among pregnant women and children, it is important to find ways to effectively communicate these risks with parents and caregivers. Thus, *PRoTECT* represents a potentially useful tool for assessing knowledge as it pertains to toxic chemicals found in the environment and their impact of children's development. In turn, parents' understanding of the impact of toxic chemicals on children's development may accelerate the promulgation of protective policies and regulations.

## Data Availability Statement

The raw data supporting the conclusions of this article will be made available upon request by the authors, without undue reservation.

## Ethics Statement

The studies involving human participants were reviewed and approved by York University Ethical Review Board. The patients/participants provided their electronic informed consent to participate in this study.

## Author Contributions

RG, BL, EP, and CT: study conception and design. RG, CG, SR, and JJ: data collection. RG, BL, CG, and DF: analysis and interpretation of results. RG and CG: draft manuscript preparation. RG, BL, EP, CG, DF, and CT: manuscript review. All authors reviewed the results and approved the final version of the manuscript.

## Funding

This work was supported by Passport Foundation, the Minderoo Foundation, and the Forsythia Foundation.

## Conflict of Interest

The authors declare that the research was conducted in the absence of any commercial or financial relationships that could be construed as a potential conflict of interest.

## Publisher's Note

All claims expressed in this article are solely those of the authors and do not necessarily represent those of their affiliated organizations, or those of the publisher, the editors and the reviewers. Any product that may be evaluated in this article, or claim that may be made by its manufacturer, is not guaranteed or endorsed by the publisher.
