# Supplementary material for: Development and Validation of the Prevention of Toxic Chemicals in the Environment for Children Tool: A Questionnaire for Examining the Community's Knowledge of and Preferences Toward Toxic Chemicals and Children's Brain Development
Source: Front Public Health. 2022 May 11;10:863071. doi: 10.3389/fpubh.2022.863071 (PMC9130721; doi:10.3389/fpubh.2022.863071)
Supplement: Supplementary file 1 [file Data_Sheet_1.pdf]

Supplemental Tables

Supplemental Table 1. *Coding matrix reflecting the number of verbal and non-verbal cues indicating agreement or dissent with items.*

Question	Consensus
**Item 1** Do you prefer: Toxic chemicals (disagree is prefer environmental chemicals)	A=11<br>D= 5<br>SE = 1<br>SD = 1<br>NU = 4<br>NR = 7
**Item 2** Do you prefer: to prevent learning and behavioural conditions (disagree is "in preventing learning and behavioural conditions")	NU = 3<br>NR = 4<br>* only asked in Focus group 1 (April 26) with 7 total participants
**Item 2** Would you like examples in brackets included in the item	A = 16<br>NR = 13
**Item 2** Would you prefer "learning and behavioural conditions"? (disagree is ADHD, autism, neurodevelopmental disorders)	D = 9<br>SD = 5<br>NR = 15
**Item 3** Do you prefer "devote"? (disagree is invest)	A = 9<br>D = 5<br>NU = 2<br>NR = 13
**Item 4** Should we remove first part of the item?	A = 12<br>SE = 1<br>D = 3<br>NR = 13
**Item 4**: If we remove the first part of the item is it leading to get an agree answer?	A = 6<br>NR = 23
**Item 5** Do you prefer "actions"? (disagree is "things")	A = 8<br>NU = 3<br>NR = 18
**Item 5** Should we phrase this item in the negative?	A = 5<br>NR = 1<br>* only asked in Focus group 4 with 6 total participants
**Item 5** Can ADHD and autism be paired once with "learning and behavioural conditions"?	A = 10<br>D = 3<br>NR = 16

**Item 6** Do you prefer "interfere"? (disagree is prefer "impact)	D = 9<br>NU = 3<br>NR = 17
**Item 6** Do you understand what the term "development" means?	A = 11<br>NR = 18
**Item 7** Do you prefer the term "budgets"? (disagree = funds/resources)	A = 7<br>NU = 1<br>NR = 21
**Item 8** Do you prefer the term "scientists"? (disagree = researchers)	A = 16<br>D = 5<br>NU = 1<br>NR = 7
**Item 8** Do you prefer the term "scientists"? (disagree = more specific type of scientist)	A = 10<br>D = 17<br>SD = 1<br>NR = 1
**Item 9** Do you prefer "before they are born"? (disagree = prenatal/gestation/in utero)	A = 2<br>D = 3<br>NR = 24
**Item 6/9** Do you know when the brain develops?	D = 8<br>NR = 21
**Item 10** Is this clear?	A = 9<br>NR = 20
**Item 11** Do you prefer "exposure to"? (disagree = more specific)	A = 15<br>D = 2<br>NR = 12
**Item 11** Do you know what "exposure to" means?	A = 6<br>D = 2<br>NU = 2<br>NR =19
**Item 12** Do you understand what this item is saying?	A = 2<br>D =7<br>NR = 20
**Item 12** Do you prefer "regulating" (disagree = reducing/reductions to)	A = 1<br>D = 7<br>SD = 1<br>NR = 20
**Item 12** Do you prefer 12 B? (disagree = 12 A)	A = 16<br>NU = 1<br>NR = 12
**Item 13** Do you prefer "want to learn more"? (disagree = "am interested in learning more")	A = 5<br>D = 1<br>SE = 1<br>NU = 4<br>NR = 18
**Item 14** Is the item clear?	A = 13

	NR = 16
**Item 15** Do you prefer “regardless”? (disagree = “no matter their)	A = 3<br>NR = 4<br>* only asked in Focus group 1 with 7 total participants
**Item 15** Do you prefer “race”? (disagree = “ethnicity”)	A = 2<br>D = 1<br>NU = 4<br>NR = 22
**Item 15** Do you prefer “opportunities” (disagree = “abilities”)	D = 4<br>NR = 25
**Item 15** Do you prefer the item worded in the negative?	A = 4<br>NU = 1<br>NR = 1<br>* only asked in Focus group 4 with 6 total participants
**Item 16** Do you like “regulations”?	A = 5<br>SE = 2<br>NR = 22
**Item 17** Do you prefer “general practitioner”? (disagree = physician/doctor/family doctor)	A = 1<br>D = 17<br>* one participant disagreed with all terms<br>NR = 10
**Item 18** Do you prefer “research”? (disagree = “studies”)	A = 7<br>D = 6<br>NR = 16
**Item 18** Do you prefer “blood”? (disagree = bodies)	A = 4<br>D = 2<br>SD = 1<br>N = 1<br>NR = 21
**Item 19** Is “after they are born” implied in the item?	A = 6<br>NR = 23

Abbreviations: A = Indicated agreement (i.e., verbal or nonverbal), D = Indicated dissent (i.e., verbal or nonverbal), SE = Provided significant statement or example suggesting agreement, SD = Provided significant statement or example suggesting dissent, NR = Did not response, NU = Indicated no preference

Supplemental Table 2. *Items in Each Round*

Item	Round 1	Round 2	Round 3

1.	Toxic chemicals in our day-to-day lives, like air pollution or lead in drinking water, can increase a child's risk of developing ADHD or autism	Toxic chemicals in our day-to-day lives, like air pollution or lead in drinking water, can increase a child's risk of developing ADHD or autism	Toxic chemicals in our day-to-day lives, like air pollution or lead in drinking water, can increase a child's risk of developing ADHD or autism
2.	The amount of resources that my government invests to prevent learning and behavioral conditions in children is about equal to the amount it invests to treat these conditions	The amount of resources that my government invests to prevent learning and behavioral conditions in children is about equal to the amount it invests to treat these conditions	Most governments invest about the same amount to prevent conditions like ADHD and autism as they spend to treat these conditions
3.	My government should devote more resources to make sure that consumer products do not contain toxic chemicals that are unsafe for children.	My government should devote more resources to make sure that consumer products do not contain toxic chemicals that are unsafe for children.	My government should strengthen their policies and programs to make sure that consumer products do not contain harmful levels of toxic chemicals.
4.	Of all the things my government does to keep children healthy, reducing children's exposure to toxic chemicals should be a priority.	Of all the things my government does to keep children healthy, reducing children's exposure to toxic chemicals should be a priority.	N/A
5.	There are things parents can do during pregnancy and early childhood to reduce their child's risk of developing a learning or behavioural condition, like ADHD or autism.	There are things parents can do during pregnancy and early childhood to reduce their child's risk of developing a learning or behavioural condition, like ADHD or autism.	N/A
6.	The levels of toxic chemicals commonly found in food, consumer products, and drinking water are too low to interfere with children's brain development.	The levels of toxic chemicals commonly found in food, consumer products, and drinking water are too low to interfere with children's brain development.	My government has effective regulations to ensure that food and personal care products do not contain harmful levels of toxic chemicals.

7.	Most governments spend 95% or more of their budgets to treat disease and disabilities. Governments should devote more of their budget to prevent these conditions	Most governments spend 95% or more of their budgets to treat disease and disabilities. Governments should devote more of their budget to prevent these conditions	Most governments spend most of their health budget to manage and treat medical conditions, including conditions like ADHD or autism. I think governments should devote more resources to find ways to prevent children from developing these condition
8.	I trust scientists' recommendations about how to reduce exposure to toxic chemicals	I trust scientists' recommendations about how to reduce exposure to toxic chemicals	Of all the sources of information about health impacts from toxic chemicals, I would trust information coming from the scientists who study them.
9.	Children are more likely to be harmed by toxic chemicals than adults, especially before they are born.	Children are more likely to be harmed by toxic chemicals than adults, especially before they are born.	Toxic chemicals are more harmful to babies and children than they are to adults.
10.	I trust companies to make products that don't contain harmful chemicals.	I trust companies to make products that don't contain harmful chemicals.	I trust most companies to make products that don’t contain harmful levels of toxic chemicals.
11.	If I knew how to reduce children's exposure to toxic chemicals, I would do it.	If I knew how to reduce children's exposure to toxic chemicals, I would do it.	If I knew how to reduce children’s exposure to toxic chemicals, I would try to do it.
12.	The number of children who would benefit from regulating toxic chemicals linked to learning and behavioural conditions is greater than the number of children who benefit from treating these conditions.	The number of children who would benefit from regulating toxic chemicals linked to learning and behavioural conditions is greater than the number of children who benefit from treating these conditions.	More children would benefit by reducing toxic chemicals linked with conditions like ADHD and autism than the number of children who benefit from treatment of these conditions
13.	I want to learn more about how to reduce children's exposure to toxic chemicals.	I want to learn more about how to reduce children's exposure to toxic chemicals.	I want to learn more about how to reduce children's exposure to toxic chemicals.

14.	Toxic chemicals are found in everyday products, including foods, cleaning products, and personal care products.	Toxic chemicals are found in everyday products, including foods, cleaning products, and personal care products.	N/A
15.	All parents have equal opportunities to protect their children from toxic chemicals, regardless of income level, race or where they live	All parents have equal opportunities to protect their children from toxic chemicals, regardless of income level, race or where they live	All parents have equal opportunities to protect their children from toxic chemicals like pesticides or heavy metals, regardless of income level, race and ethnicity, or where they live.
16.	My government has regulations to make sure that personal care products, furnishings, and food do not contain harmful levels of toxic chemicals.	My government has regulations to make sure that personal care products, furnishings, and food do not contain harmful levels of toxic chemicals.	My government has effective regulations to ensure that food and personal care products do not contain harmful levels of toxic chemicals
17.	If toxic chemicals were a threat to my family's health, my pediatrician, obstetrician, or general practitioner would have told me about it.	If toxic chemicals were a threat to my family's health, my pediatrician, obstetrician, or general practitioner would have told me about it.	If toxic chemicals were a threat to my family’s health, my pediatrician, doctor, or health care provider would have told me about it.
18.	Research shows that most pregnant women have toxic chemicals in their blood	Research shows that most pregnant women have toxic chemicals in their blood	Toxic chemicals can be detected in the blood of most pregnant women.
19.	N/A	Toxic chemicals that pregnant women are exposed to can increase the risk of their child having a learning or behavioural condition after they are born.	Toxic chemicals found in pregnant women can increase their child’s risk of having a learning or behavioural condition.
20.	N/A	N/A	I try to purchase products that do not contain toxic chemicals that may be harmful to my family

Supplemental Table 3. *Items clustered by factor*.

Factor 1: Desire to reduce exposure to toxic chemicals
Of all the sources of information about health impacts from toxic chemicals, I trust information coming from scientists who study them.

More children would benefit by regulating and reducing toxic chemicals to ***prevent*** *developmental conditions than the number of children who benefit from* ***treatment*** *of these conditions.*

Toxic chemicals can be detected in the blood of most pregnant women.

My government should strengthen their policies and programs to make sure that consumer products do not contain toxic chemicals that are harmful to children.

If I knew how to reduce children's exposure to toxic chemicals, I would try to do it.

I try to purchase products that do not contain toxic chemicals that may be harmful to my family.

*I am worried that my family may be exposed to toxic chemicals.**

I want to learn more about how to reduce children's exposure to toxic chemicals.

Factor 2: Trust in sources of toxic chemicals

My government has effective regulations to ensure that food and personal care products do not contain harmful levels of toxic chemicals.

If toxic chemicals were a threat to my family's health, my pediatrician, doctor, or health care provider would have told me about it.

*I am worried that my family may be exposed to toxic chemicals.**

Factor 3: Knowledge of developmental toxicity

Toxic chemicals in our day-to-day lives, like air pollution or lead in drinking water, can increase a child's risk of developing conditions like ADHD or autism.

Reducing exposure to toxic chemicals during pregnancy and in early childhood can help lower a child's risk of developing a condition like ADHD or autism.

When it comes to addressing developmental conditions affecting children, most governments spend the majority of the health budget on management and treatment of these conditions. I think governments should spend more of their budget to find ways to prevent children from developing these conditions.

Exposure to toxic chemicals during pregnancy can increase a child's risk of having a developmental condition.

Factor 4: Knowledge of government and society about toxic chemicals and exposure

---

Most governments invest about the same amount to ***prevent*** *developmental conditions as they spend to* ***treat*** *these conditions.*

All parents have equal opportunities to protect their children from toxic chemicals like pesticides or heavy metals, regardless of income level, race and ethnicity, or where they live.

**Equal loading onto two factors.* **No loading: *"Toxic chemicals are generally more harmful to babies and children than they are to adults."*

Supplemental Figures

Supplemental Figure 1. *PRoTECT Version used for EFA*

PROTECT

PRevention Of Toxic chemicals in the Environment for Children Tool

PROTECT is a survey to find out what parents and people of childbearing age know about toxic chemicals and developmental conditions in children. Toxic chemicals, like lead, pesticides and phthalates, can be found in our homes, air, and water, and can be harmful to our health. Developmental conditions include learning disabilities, attention deficit/hyperactivity disorder (ADHD), and autism spectrum disorder (autism), among other developmental conditions.

We are asking you to rate each item on a scale from "Strongly Agree" to "Strongly Disagree". If you come to an item that you are unsure of the answer, please give your best guess and do not skip any items.

	1 – Strongly Agree	2 – Somewhat Agree	3 – Neither Agree nor Disagree	4 – Somewhat Disagree	5 – Strongly Disagree
1. Toxic chemicals in our day-to-day lives, like air pollution or lead in drinking water, can increase a child’s risk of developing conditions like ADHD or autism.	**1**	**2**	**3**	**4**	**5**
2. Most governments invest about the same amount to **prevent** developmental conditions as they spend to **treat** these conditions.	**1**	**2**	**3**	**4**	**5**
3. All parents have equal opportunities to protect their children from toxic chemicals like pesticides or heavy metals, regardless of income level, race and ethnicity, or where they live.	**1**	**2**	**3**	**4**	**5**
4. My government has effective regulations to ensure that food and personal care products do not contain harmful levels of toxic chemicals.	**1**	**2**	**3**	**4**	**5**

5. Reducing exposure to toxic chemicals during pregnancy and in early childhood can help lower a child's risk of developing a condition like ADHD or autism.	**1**	**2**	**3**	**4**	**5**
6. When it comes to addressing developmental conditions affecting children, most governments spend the majority of the health budget on management and treatment of these conditions. I think governments should spend more of their budget to find ways to prevent children from developing these conditions.	**1**	**2**	**3**	**4**	**5**
7. If toxic chemicals were a threat to my family's health, my pediatrician, doctor, or health care provider would have told me about it.	**1**	**2**	**3**	**4**	**5**
	1 – Strongly Agree	2 – Somewhat Agree	3 – Neither Agree nor Disagree	4 – Somewhat Disagree	5 – Strongly Disagree
8. Exposure to toxic chemicals during pregnancy can increase a child's risk of having a developmental condition	**1**	**2**	**3**	**4**	**5**
9. I want to learn more about how to reduce children's exposure to toxic chemicals.	**1**	**2**	**3**	**4**	**5**
10. Of all the sources of information about health impacts from toxic chemicals, I trust information coming from scientists who study them.	**1**	**2**	**3**	**4**	**5**
11. Toxic chemicals are generally more harmful to babies and children than they are to adults.	**1**	**2**	**3**	**4**	**5**
12. I trust that most companies make products that don't contain harmful levels of toxic chemicals.	**1**	**2**	**3**	**4**	**5**
13. More children would benefit by regulating and reducing toxic chemicals to **prevent** developmental conditions than the number of children who benefit from **treatment** of these conditions.	**1**	**2**	**3**	**4**	**5**
14. Toxic chemicals can be detected in the blood of most pregnant women.	**1**	**2**	**3**	**4**	**5**
15. My government should strengthen their policies and programs to make sure that consumer products do not contain toxic chemicals that are harmful to children.	**1**	**2**	**3**	**4**	**5**
16. If I knew how to reduce children's exposure to toxic chemicals, I would try to do it.	**1**	**2**	**3**	**4**	**5**

17. I try to purchase products that do not contain toxic chemicals that may be harmful to my family.	**1**	**2**	**3**	**4**	**5**
18. I am worried that my family may be exposed to toxic chemicals.	**1**	**2**	**3**	**4**	**5**
